# Current Advances in Japanese Encephalitis Virus Drug Development

**DOI:** 10.3390/v16020202

**Published:** 2024-01-28

**Authors:** Jiao Guo, Yunqi Mi, Yan Guo, Yang Bai, Meihua Wang, Wei Wang, Yang Wang

**Affiliations:** 1 The Xi’an Key Laboratory of Pathogenic Microorganism and Tumor Immunity, School of Basic Medicine, Xi’an Medical University, Xi’an 710021, China; guojiao@xiyi.edu.cn (J.G.); 30111205513@stu.xiyi.edu.cn (Y.M.); 30111205508@stu.xiyi.edu.cn (Y.B.); 2 College of Animal Science and Technology, College of Veterinary Medicine, Huazhong Agricultural University, Wuhan 430070, China; guoyan@webmail.hzau.edu.cn; 3 Faculty of Life Science and Medicine, University of Science and Technology of China, Hefei 230026, China; wmeihua@ustc.edu.cn; 4 State Key Laboratory of Virology, Wuhan Institute of Virology, Chinese Academy of Sciences, Wuhan 430071, China

**Keywords:** JEV, flaviviruses, antiviral drug development

## Abstract

Japanese encephalitis virus (JEV) belongs to the *Flaviviridae* family and is a representative mosquito-borne flavivirus responsible for acute encephalitis and meningitis in humans. Despite the availability of vaccines, JEV remains a major public health threat with the potential to spread globally. According to the World Health Organization (WHO), there are an estimated 69,000 cases of JE each year, and this figure is probably an underestimate. The majority of JE victims are children in endemic areas, and almost half of the surviving patients have motor or cognitive sequelae. Thus, the absence of a clinically approved drug for the treatment of JE defines an urgent medical need. Recently, several promising and potential drug candidates were reported through drug repurposing studies, high-throughput drug library screening, and de novo design. This review focuses on the historical aspects of JEV, the biology of JEV replication, targets for therapeutic strategies, a target product profile, and drug development initiatives.

## 1. Introduction

JEV is a typical zoonotic, mosquito-borne flavivirus of the *Flaviviridade* family, which also includes other important mosquito-borne pathogens such as dengue virus (DENV), yellow fever virus (YFV), West Nile virus (WNV), Zika virus (ZIKV), and tick-borne encephalitis virus (TBEV) [1,2]. The virus can be transmitted by the *Culex tritaeniorhynchus* or *Culex vishnui* mosquito via waterfowl and pigs, with humans becoming infected when bitten by the infected mosquito vector [3,4]. JEV can cross the blood–brain barrier (BBB) and enter the central nervous system (CNS), resulting in a strong inflammatory response and neuronal death [3,5]. Laboratory diagnosis of JE is achieved via a JEV-specific immunoglobulin M (IgM)-capture enzyme-linked immunosorbent assay (ELISA), which has high levels of specificity and sensitivity [3,6,7]. As a pre-travel precaution in most countries, multiple doses of JEV vaccines are recommended [8]. Vaccination is considered a dependable means of prevention, in addition to avoiding mosquito bites [9]. A live attenuated vaccine (SA14-14-2) and purified formalin-inactivated mouse brain vaccine are available; the former has been shown to be effective, safe, and inexpensive [3]. To date, there is no specific clinically approved treatment for JEV infection; only symptomatic treatments are available, highlighting the urgency and importance of developing effective therapeutics [10,11]. 

## 2. Global Distribution of JEV 

**Figure 1:**
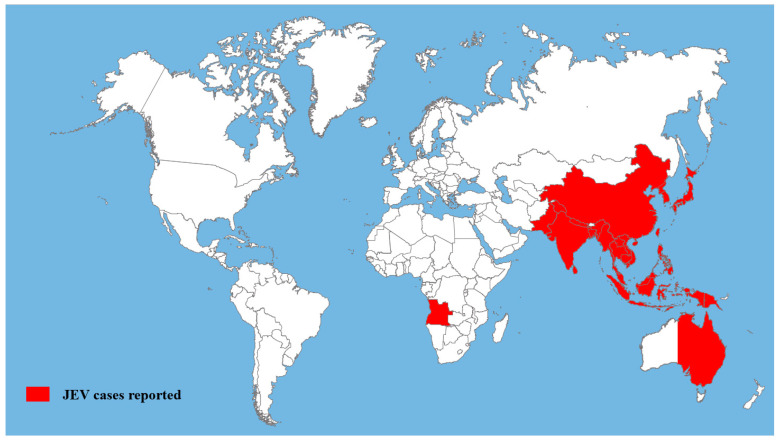
Geographical distribution of JEV infection. Map reproduced from Srivastava et al., 2023 [14], Sharma et al., 2021 [19], Mackenzie et al., 2022 [11], van den Hurk et al., 2009 [12], and Mulvey et al., 2021 [25].

Until recently, the distribution of JEV was steadily increasing [12,13,14]. The first case of JEV was reported in Japan in 1871, and the first prototype strain, Nakayama, was isolated from the brain of a fatal case in 1935 [11,15]. JEV is documented to have originated from an ancestral virus in the Indonesia–Malaysia area and may have evolved over the past 300 years, diverging into genotypes I-V via nucleotide sequencing of the C/PrM and E genes and spreading throughout Asia and Australia [11,15,16,17]. JE cases have been reported mainly from Southeast Asian countries such as Nepal, India, Burma, Bangladesh, China, Vietnam, Thailand, the Philippines, Indonesia, and Malaysia (Figure 1) [11,14,18,19]. Long-distance mosquito movement can occur through abiotic mechanisms, such as commercial air or sea transport. This may have been the case in the spread of JEV to Angola [20]. Genotypes I and III were mainly distributed in temperate areas of Asia, while genotypes II and IV were mostly associated with epidemic diseases in tropical regions [21,22]. Genotype V was restricted to Malaysia, but isolated cases have recently been reported in China and the Republic of Korea [23,24]. 

## 3. Molecular Biology of JEV

**Figure 2:**
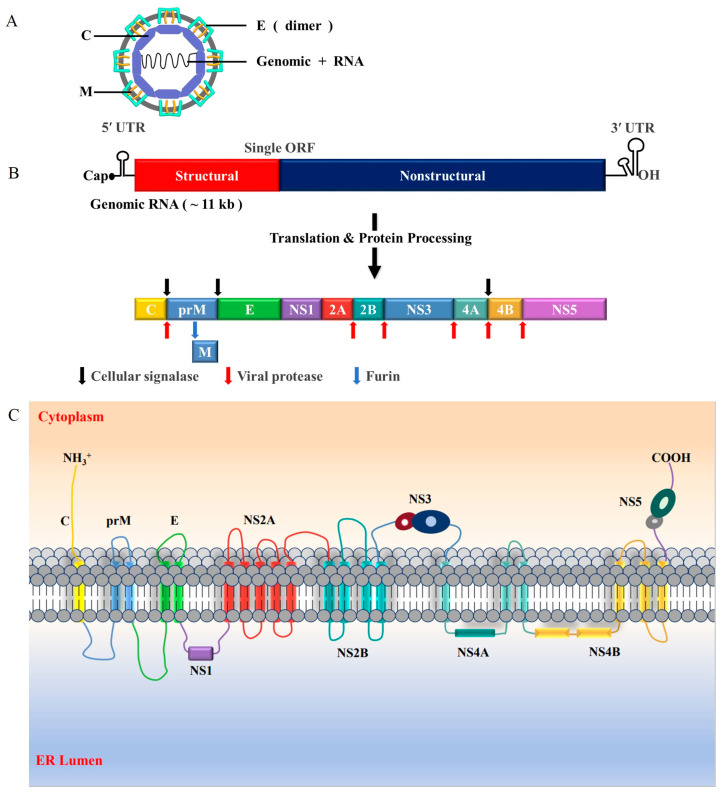
JEV structural organisation and genome representation. (**A**) The nucleocapsid (genomic RNA within a viral capsid protein shell) is surrounded by a lipid bilayer containing viral proteins M and E. (**B**) The JEV genome is approximately 11 kb in length, is capped at the 5′ end, and has no 3′ poly (**A**) tail. Viral structural genes (represented by a red rectangle) and non-structural genes (represented by a blue rectangle) encoded in the (+)-strand RNA genome are shown. The viral proteins (C, PrM, E, NS1, NS2A, NS2B, NS3, NS4A, NS4B, and NS5) are produced by processing the polyprotein using either a cellular signalase (indicated by black arrows) or the viral NS2A/NS3 protease (represented by red arrows). The PrM protein undergoes further processing by host furin (represented by a blue arrow) to generate glycosylated M protein, a constituent of the flavivirus mature virion. (**C**) A diagram showing the arrangement of the JEV polyprotein in the ER membrane is presented. The arrangement of JEV’s structural and non-structural proteins in relation to the cytosol and ER lumen is illustrated. The proteins of JEV are distributed in the cytoplasm (NS3 and NS5), in the ER lumen (NS1), and in the ER membrane (C, PrM/M, E, NS2A, NS2B, NS4A, and NS4B). The figure is adapted from Ray et al., 2006 [55], Sharma et al., 2021 [19], Kumar et al., 2022 [56], and Pierson et al., 2020 [39].

JEV is a small, icosahedral enveloped virus with a diameter of 50 nm [26]. It has an approximately 11 kb positive-sense viral RNA genome containing a single open reading frame (ORF) flanked by untranslated regions (UTRs) at both termini (Figure 2) [19]. The ORF encodes a polyprotein that is further cleaved by host and viral proteases into ten proteins, three of which are structural proteins named capsid (C), pre-membrane (PrM), and envelope (E), and the remaining seven are non-structural (NS) proteins named NS1, NS2A, NS2B, NS3, NS4A, NS4B, and NS5 [2,10]. The UTRs comprise RNA elements crucial for the efficient translation and replication of the JEV genome [27]. A type-1 cap structure (m^7^GpppA^m^p) is present at the 5’end of the genome, while a CU_OH_ (instead of a poly (A) tail) terminates the 3′ end [27].

The C protein is a highly basic protein that has an affinity for viral RNA and lipid membranes and forms part of the viral nucleocapsid [28,29,30]. The E protein is a glycoprotein that forms the shell of flaviviruses and plays a key role in viral attachment and fusion [31,32]. The PrM is a glycoprotein that facilitates E protein folding and regulates the oligomeric state of E proteins to prevent adventitious fusion during the release of virus particles from infected cells [32,33,34]. The maturation of the viral particle occurs through the protease hydrolysis of the PrM, resulting in the formation of the M protein [35]. A near-atomic structure of JEV has confirmed several structural determinants associated with viral neurovirulence and stability [36], and the PrM/E protein is the critical virulence determinant of JEV [37]. 

The seven non-structural proteins are a critical part of the viral replication complex and interact with host proteins; they participate in viral replication, virion assembly, and virus escape from immune surveillance [38,39,40]. NS1 is a glycoprotein and is generally localised with double-stranded RNA (dsRNA); it may interact with the transmembrane proteins NS4A and NS4B [41,42]. NS1 exists in several oligomeric forms and in membrane-bound forms in the endoplasmic reticulum (ER) as a component of a larger viral replication complex [43,44]. Secreted NS1 serves as a diagnostic marker for the initial phases of human flavivirus infection in the bloodstream, where immune system proteins encounter the sNS1 hexamer as a proteolipid particle [40,45,46]. NS2A is critical for genome synthesis and assembly [47]. NS2B forms a protease complex with NS3 and exhibits N-terminal serine protease activity [48,49]. NS4A is regarded as a crucial “organiser” of the flavivirus replication complex and consists of a C-terminal fragment called 2K, a hydrophilic N-terminal protein located in the cytoplasm, and three internal hydrophobic regions associated with ER membranes (pTMS1–pTMS3) [50,51,52,53]. The role of the JEV NS4B protein is unclear; studies on flaviviruses showed that NS4B is the largest hydrophobic non-structural protein of flaviviruses and contains two hydrophobic segments (pTMD1 and pTMD2) and three C-terminal transmembrane segments (pTMD3–pTMD5) [54].

Among the proteins, NS3 and NS5 have enzymatic activities required for viral RNA synthesis [57]. NS3 is a multifunctional protein with a proteolytic domain at its N-terminus, and its C-terminal region exhibits helicase activity; therefore, it is significantly involved in the processing of viral polyprotein and replication by controlling the activities of proteases, helicases, and nucleoside 5′-triphosphatase [58,59]. NS5 is the largest and most highly conserved multi-enzymatic flaviviral protein containing a C-terminal RNA-dependent RNA polymerase (RdRp) domain and an N-terminal methyltransferase (MTase) domain [60,61]. Variations in NS5 have been shown to decrease IFN-α and β production more efficiently in different JEV strains due to their advantage in host adaptation [62]. NS5 has also been shown to damage host lipid metabolism to enhance the proinflammatory response, resulting in increased neurovirulence and neuroinvasiveness in vivo [63], and it is the most potent and direct antagonist of the IFN-I-dependent JAK-STAT signalling pathway encoded by all flaviviruses studied to date [60,64]. The importance of NS5′s function in IFN-I antagonism and flavivirus resistance to IFN-1 demonstrate that NS5 is certainly one of the determinants of the potential of flaviviruses to emerge in humans [65,66]. With the availability of high-resolution crystal structures, the NS5 protein domains of flaviviruses are attractive targets for the discovery of direct-acting antiviral agents [60,67].

## 4. Life Cycle of JEV

**Figure 3:**
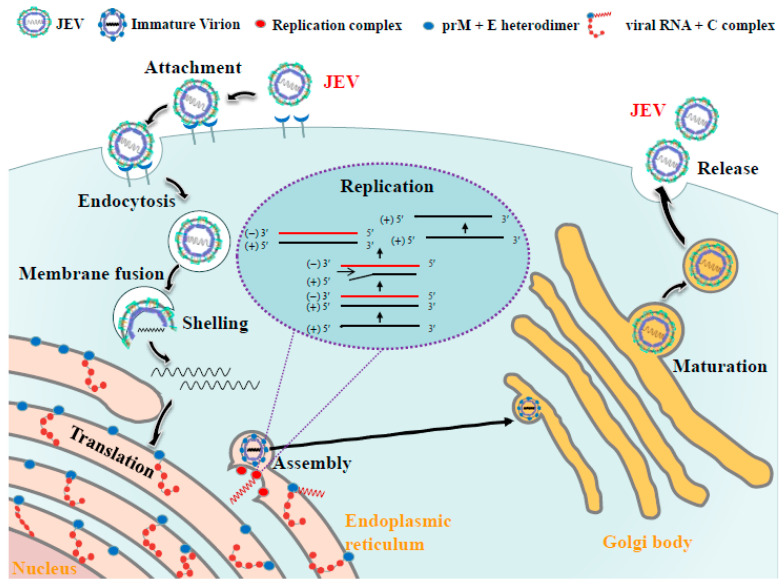
JEV life cycle. Virions are adsorbed onto the host cellular membrane and then enter the cell via receptor-mediated endocytosis. A low-pH environment in the endosome initiates the rearrangement of the viral envelope, culminating in membrane fusion and the release of the genome into the cytoplasm. The positive-sense viral RNA is translated, resulting in a single polyprotein that is cleaved into structural and non-structural proteins. Viral replication is facilitated by a specialized compartment (comprising NS5, the RdRp, and other viral non-structural proteins and multiple host factors). This compartment transcribes the positive-strand genomic RNA into a negative-strand RNA, which is then employed as a template to produce progeny (+)-strand genomes. After replication, the genome of the virus is encapsidated by C protein and transported to the ER, where the nucleocapsid is enveloped by a lipid bilayer in which the PrM and E proteins are embedded. Immature virions, consisting of genomic RNA, PrM-E heterodimers, and C, travel through the trans-Golgi network and mature via furin cleavage. This figure was originally created using BioRender.com, accessed on 20 October 2023.

Like other known flaviviruses, the life cycle of JEV can be roughly divided into stages of binding, entry, translation, replication, assembly, and release (Figure 3). Upon infection, JEV enters the host cell via receptor-mediated endocytosis [10,68,69]. Subsequently, the E protein binds with the endosomal membrane and releases the viral RNA genome into the cytoplasm [70,71]. The positive-sense viral RNA acts as mRNA and is translated to generate a polyprotein. This polyprotein is cleaved into structural and non-structural proteins by both the viral NS2B-NS3 protease and the host proteases [10]. The NS proteins form a viral replication complex with viral RNA on the ER membrane and synthesise multiple copies of viral RNA and unknown host proteins [21,72]. The C protein binds to viral RNA to form a nucleocapsid, which subsequently acquires a lipid envelope consisting of the membrane and envelope proteins to form immature virion particles [30]. The immature virus particles travel through the trans-Golgi network and undergo a maturation process through furin cleavage [34]. E proteins on the surface of the virus particles rearrange into a herringbone pattern that tiles the surface of the virus particle [34,73]. The mature particles then exit the cell through exocytosis [3,16]. 

## 5. Target Product Profile (TPP)

No definitive TPP for the development of therapeutics for JEV infection is currently available from the WHO. Our laboratory provides an overview of possible challenges and requirements for the treatment of JEV infection. A critical primary point to include in the TPP for JEV therapeutics is that drugs for acute infection must cross the BBB to reach the ultimate target cells of viral infection. In addition, any drug used to treat pregnant women should have a high safety profile to avoid the risk of harm to the foetus. A comprehensive description of our hypothetical TPP is shown in Table 1.

## 6. Drug Development

### 6.1. Entry Inhibitors

The identification of host and viral factors involved in JEV entry has been an active area of scientific investigation in the literature. Many different molecules have been demonstrated to interact with JEV on the cell surface, and several studies have identified potential receptors such as heat shock protein, glucose-regulated protein 78 (GRP78), C-type lectin member 5A (CLEC5A), T-cell immunoglobulin and mucin domain 1 (TIM-1), dendritic cell-specific intercellular adhesion molecule-3-grabbing non-integrin (DC-SIGN), vimentin, laminin receptor, and CD4 in different cell types [10,19,74,75,76,77,78,79,80]. 

**Figure 4:**
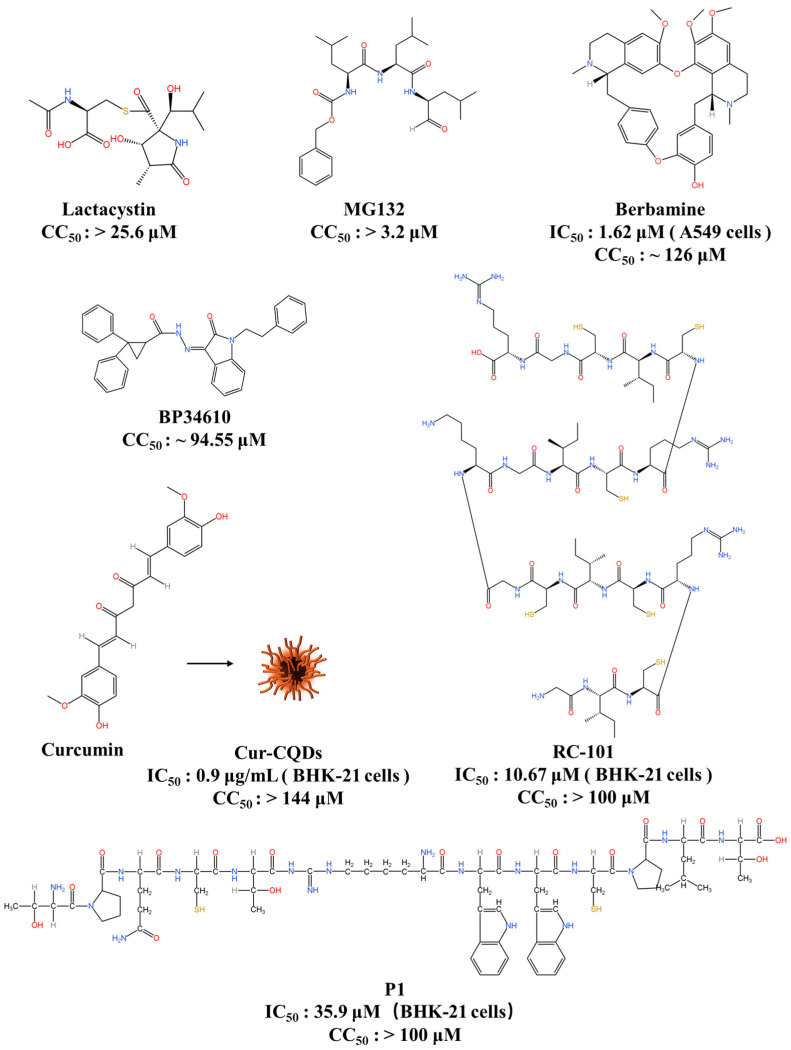
JEV entry inhibitors. This figure was originally created using KingDraw software 3.1.0.20.

The JEV E protein is a class II viral fusion protein that mediates host cell entry, making interference with E protein–host receptor interactions an attractive strategy for JEV drug development [3,31]. Interfering with viral entry into host cells using peptides targeting viral surface proteins has been shown to be an effective strategy for inhibiting viral infection [81]. Phage display is a potent method of identifying special molecules that exhibit specific affinities towards a specific target from a vast range of libraries [82]. Wei et al. demonstrated that that a peptide (designated P1) derived from the Ph.D.-12^TM^ phage display peptide library against the JEV E protein, with an amino acid sequence of TPDCTRWWCPLT, has the potential to inhibit JEV infection in BHK-21 cells, with an 50% inhibitory concentration (IC_50_) of 35.9 μM and low cytotoxicity (Figure 4). A BiFC assay suggests that P1 directly interacts with the JEV E protein [83]. In a lethal mouse model of JEV infection, the administration of P1 protected 3-week-old female C57BL/6 mice (5 mg/kg of P1 intraperitoneally once daily for 21 days), reduced histopathological damage and viral burden in their brains, and significantly decreased mortality rates. The treatment group showed fewer adverse effects compared to controls. This suggests that optimizing the pharmacokinetic properties of P1 may yield a potential drug candidate. 

Recently, the low-density lipoprotein receptor (LDLR), a single-chain transmembrane glycoprotein, was identified as a host factor necessary for JEV entry [84,85]. Berbamine, derived from herbs, is an ATP-competitive inhibitor of Ca^2+^/calmodulin-dependent protein kinase II (CaMKII) [86,87]. It has been found to block the entry of JEV by inhibiting the level of LDLR at the plasma membrane. The selectivity index (SI) value of berbamine is approximately 78, which suggests its good therapeutic window as an antiviral agent against JEV [84]. Furthermore, the administration of berbamine at a dosage of 15 mg/kg via intraperitoneal injection twice daily for a span of 14 days resulted in the protection of 3–4-week-old BALB/c mice against a lethal JEV challenge, which is evidenced by an improved rate of survival—75% in the group treated with berbamine compared to 12.5% in the control group. This finding suggests that berbamine holds great therapeutic potential as an antiviral agent for JEV.

During cell-based high-throughput screening that utilized dengue reporter viruses, BP34610, a new flavivirus entry inhibitor, was discovered. This inhibitor effectively suppressed both DENV and JEV viral yields without causing detectable cytotoxicity. By utilizing a plaque reduction assay, researchers found that at 5 μM, BP34610 could inhibit 99% of the viral yield of JEV yield [88]. This result suggests that BP34610 is a broad-spectrum flavivirus inhibitor that may target the flavivirus E protein and it also provides valuable insight into the intricate mechanisms governing flavivirus infection. 

Retrocyclin is a circular θ-defensin peptide which has been artificially humanised and was previously reported to have broad antimicrobial activity [89,90,91,92]. Recent research has shown that the novel θ-defensin retrocyclin-101 (RC-101) has inhibitory activity against JEV infection. In BHK-21 cells, RC-101 blocks flavivirus entry by targeting the DE loop of the E glycoprotein and has an IC_50_ of 10.67 μM [38]. This study provides a basis for the development of RC-101 as a potential strategy for treating JEV infection. 

The ubiquitin–proteasome system (UPS), which is responsible for intracellular protein degradation, plays a pivotal role in numerous cellular processes, including apoptosis, the cell cycle, endocytosis, the host immune response, and signal transduction [93,94]. Wang et al. discovered that UPS facilitates JEV entry and identified two proteasome inhibitors (MG132 and lactacystin) that hindered JEV’s intracellular fate, from cell membrane penetration to initial translation. These findings establish the role of UPS in productive JEV internalization and provide a better understanding of the interaction between the virus and the host during the early stages of infection, which may lead to the discovery of new therapeutic targets [95].

The antiviral and anti-inflammatory compound curcumin (Cur), which originates from the roots of Curcuma longa, faces limitations in biomedical research due to its elevated cytotoxicity and extremely low solubility [96,97]. Chen et al. produced low-cytotoxicity carbon-based nanomaterials, cur carbon quantum dots (Cur-CQDs), via mild pyrolysis-induced polymerisation and carbonisation [98]. The researchers discovered that the Cur-CQDs (IC_50_ = 0.9 μg/mL) strongly suppress JEV infection in BHK-21 cells in a concentration-dependent manner. The findings indicate that Cur-CQDs can effectively attach to the JEV surface and prevent its attachment and/or entry into the host cell by binding to the E-S123/K312 sites of the E protein. Furthermore, the antiviral effect of Cur-CQDs on DENV-2 and enterovirus 71 (EV71) suggests the potential to use Cur-based carbon nanomaterials to inhibit a variety of viral infections [98,99].

Due to the limited scientific understanding of JEV entry mechanisms, including molecular requirements and pathways, it is necessary to identify additional targets to validate the anti-infective mechanisms of the compounds mentioned above. Moreover, animal studies should be thoroughly evaluated for these potential leads. Furthermore, a deeper understanding of the mechanism of action of the above drugs will help us to understand the biological events of JEV entry into cells.

### 6.2. RdRp Inhibitors

As human cells do not possess RNA-dependent DNA or RNA polymerases, this specific enzyme class presents an enticing target for antiviral medications against viruses that utilize polymerases for replication. Nucleoside analogues could be utilized to target viral polymerase activity [100,101]. Several nucleosides and/or their prodrugs have demonstrated potent antiviral effects against diverse virus families in cell-based assays and animal models of infection. Examples include emtricitabine and tenofovir disoproxil fumarate for human immunodeficiency virus (HIV) [102], sofosbuvir for the hepatitis C virus (HCV) [103] and ZIKV [104], and aciclovir and penciclovir for herpes simplex virus (HSV) [105]. These compounds are promising candidates for the clinical treatment of viral infections due to their efficacy and safety. They serve as a useful reference and direction for the development of JEV antiviral drugs. 

**Figure 5:**
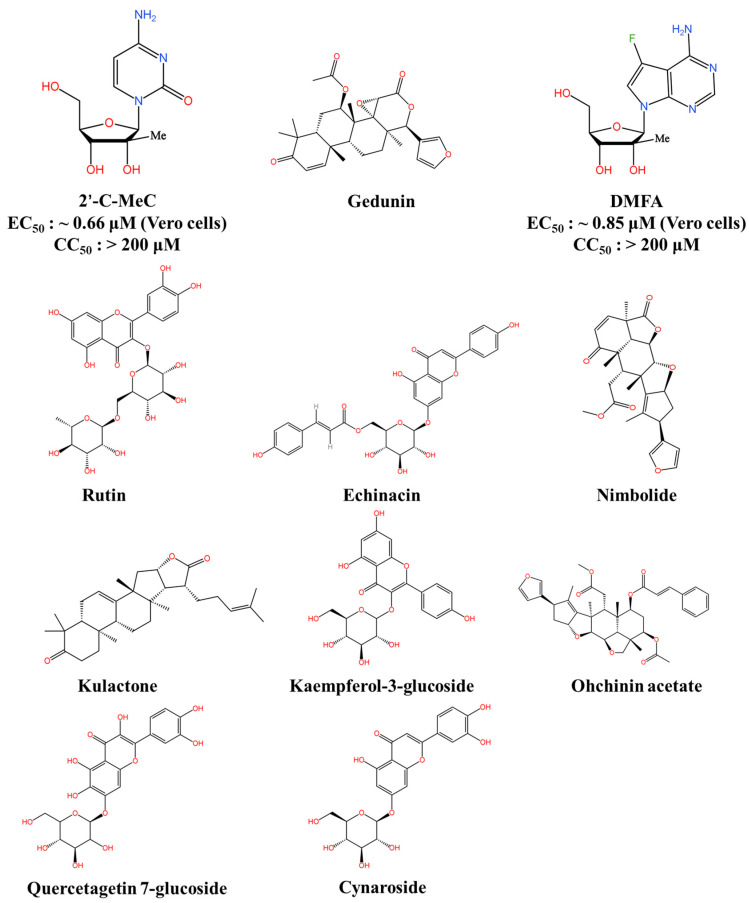
JEV RdRp inhibitors. This figure was originally created using KingDraw software 3.1.0.20.

The RdRps of the flaviviruses are similar, with JEV and WNV sharing 70% identity with ZIKV, whereas DENV-2 and DENV-3 share 76% and 81% identity, respectively [106,107]. Zandi and colleagues have developed a molecular model of the RdRp replication complex for JEV and DENV. They found that two nucleoside analogues, namely 2′-C-methyl-cytidine (2′-C-MeC) and 7-deaza-7-fluoro-2′-C-methyl-adenosine (DFMA), demonstrated potent and selective anti-JEV and anti-DENV properties with low toxicity in a cell-based assay system (Figure 5) [108]. A replicon assay was conducted to objectively evaluate the inhibitory effects of 2′-C-MeC and DFMA on JEV replication in Vero cells. The 50% effective concentration (EC_50_) values for 2′-C-MeC and DFMA were approximately 0.66 μM and 0.85 μM, respectively. The findings offer a logical structure and rationale for discovering direct-acting antiviral compounds capable of dual activity against JEV and DENV infections. 

Recently, a study employed natural products from *E. angustifolia* as ligand libraries for a structure-based virtual screening against the nucleotide GTP-binding pocket in the JEV RdRp’s crystal structure (PDB ID: 4HDG) [109]. Based on a significant docking energy (>−10 kcal/Mol) and a pharmacokinetic analysis, they identified six top-docked poses of compounds: echinacoside, echinacin, rutin, cynaroside, quercetagetin 7-glucoside, and kaempferol-3-glucoside. The redocking analysis showed that these poses each had a significant binding score, which helped to determine their similarity properties and ideal docking conformation. Thus, the six compounds derived from *E. angustifolia* demonstrate the potential to inhibit the JEV RdRp, indicating the possibility of developing highly effective antiviral drugs against Japanese encephalitis (JE).

Dwivedi et al. employed the MTiOpenScreen server to conduct a structure-based virtual screening [110]. Their results predicted the antiviral activity of gedunin, nimbolide, ohchinin acetate, and kulactone against the JEV RdRp. Of these tested compounds, four had noteworthy docking scores and druglikeness. However, their low BBB penetration may impact their efficacy. Additional in vitro and in vivo experiments are required to identify potential treatments for JEV.

### 6.3. Protease Inhibitors

Of all the viral targets, the flavivirus NS2B-NS3 protease has been one of the most actively studied due to its critical role in viral replication and maturation [111]. The JEV NS2B-NS3 serine protease plays a key role in the cytoplasmic cleavage events that occur during viral polyprotein maturation [49,54]. In view of that, NS3 protease inhibition of functionally equivalent proteins in HCV has been demonstrated to serve as an effective antiviral treatment approach during the clinical management of the infection [112]. Currently available crystal structures of free or inhibitor-bound flavivirus NS2B-NS3 protease could be utilized for drug design and development [113,114]. Peptidomimetic compounds and substrate mimic peptides are potential drug categories for creating new blockers against the JEV NS2B-NS3 protease, taking into account the triumph of protease inhibitors in treating HCV and HIV.

**Figure 6:**
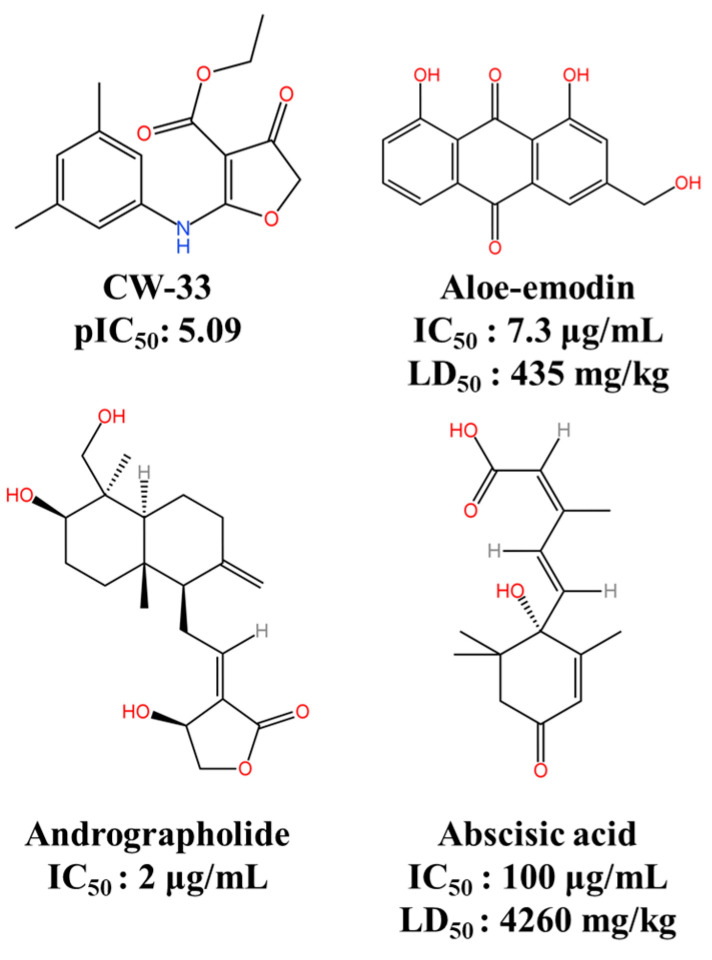
JEV protease inhibitors. This figure was originally created using KingDraw software 3.1.0.20.

One novel compound is CW-33 (ethyl 2-(3′,5′-dimethylanilino)-4-oxo-4,5-dihydrofuran-3-carboxylate), which acts as an inhibitor displaying inhibitory activity against JEV. With IC_50_ values ranging from 12.7 to 38.5 μM, this compound was tested using the supernatant virus yield assay [115]. Chen et al. conducted a study on the JEV NS2B-NS3 protease and CW-33 through a molecular docking simulation, which revealed that ligand–protein interactions were linked to the antiviral properties of CW-33 (Figure 6). The study utilized multiple linear regression (MLR) and support vector machine (SVM) models to obtain the experimental and predicted pIC_50_ value of CW-33, which was 5.09. The findings illustrate the necessary structural features for CW-33 to bind with the JEV NS2B-NS3 protease and have potential applications in therapeutic development for JEV [116].

Recently, molecular docking has emerged as a prevalent computational method for forecasting a ligand’s preferred configuration in a target’s active site. Shailesh and colleagues utilised Schrodinger suite 2019-3 to ascertain that andrographolide reacted favourably with the NS3 protease of JEV by having a good binding affinity through hydrogen bonding with LYS 73 and ASN 152 [117]. The testing of the compound in an in vitro target-based assay indicated that the molecule inhibited the NS3 protease in a concentration-dependent manner, with an IC_50_ value of 2 μg/mL. These findings suggest that andrographolide has the potential to become a potent anti-JEV agent.

A study assessed abscisic acid and aloe-emodin’s antiviral potential against the JEV NS2B-NS3 protease using a computational and target-based assay in a concentration-dependent manner [48]. The IC_50_ values for abscisic acid and aloe-emodin were found to be 100 μg/mL and 7.3 μg/mL, respectively. In addition, abscisic acid exhibited non-hepatotoxic, non-carcinogenic, non-mutagenic, non-immunogenic, and non-cytotoxic properties according to the toxicity prediction results. However, aloe-emodin was forecasted to have an inferior safety profile compared to abscisic acid, as it was anticipated to exhibit cytotoxic and mutagenic properties. As a result, additional adequate experimental studies are required to confirm the inhibitory effect of aloe-emodin against JEV, along with its safety profile. Importantly, aloe-emodin is a natural anthraquinone derivative found in the rhizome and root of Rheum palmatum [118]. It has demonstrated inhibition of the replication of EV71 [119], human cytomegalovirus (HCMV) [120], HSV [120], and influenza virus (IAV) [121]. These findings suggest a potential starting point for the development of novel broad-spectrum antivirals based on anthraquinone [118].

Novel chemical entities and the exploration of existing molecules are necessary. Computational techniques are highly beneficial during the early stages of development, aiding in the prediction of pharmacokinetics and safety parameters. Computer-aided drug design could improve the odds of identifying suitable candidates by identifying critical compound features required for the drug development process and accelerating drug research. However, this approach has limitations as the docking simulation assay was inappropriate for investigating the antiviral activities of compounds. Currently, this remains at the theoretical design and analysis stage, and it is crucial to conduct in vivo and in vitro experiments to determine the antiviral activities of compounds in future studies.

### 6.4. Bioactive Natural Products and Their Derivatives

Bioactive natural products are invaluable offerings from nature to humanity, providing inspiration for novel concepts, techniques, and remedies to combat pathogens, owing to their extensive history of use in the treatment of both epidemic and endemic diseases [122,123]. Bioactive natural products and their derivatives are crucial resources for antiviral drug discovery. Here, we draw attention to natural products with various structural backbones which have demonstrated significant antiviral properties against JEV infection. 

**Figure 7:**
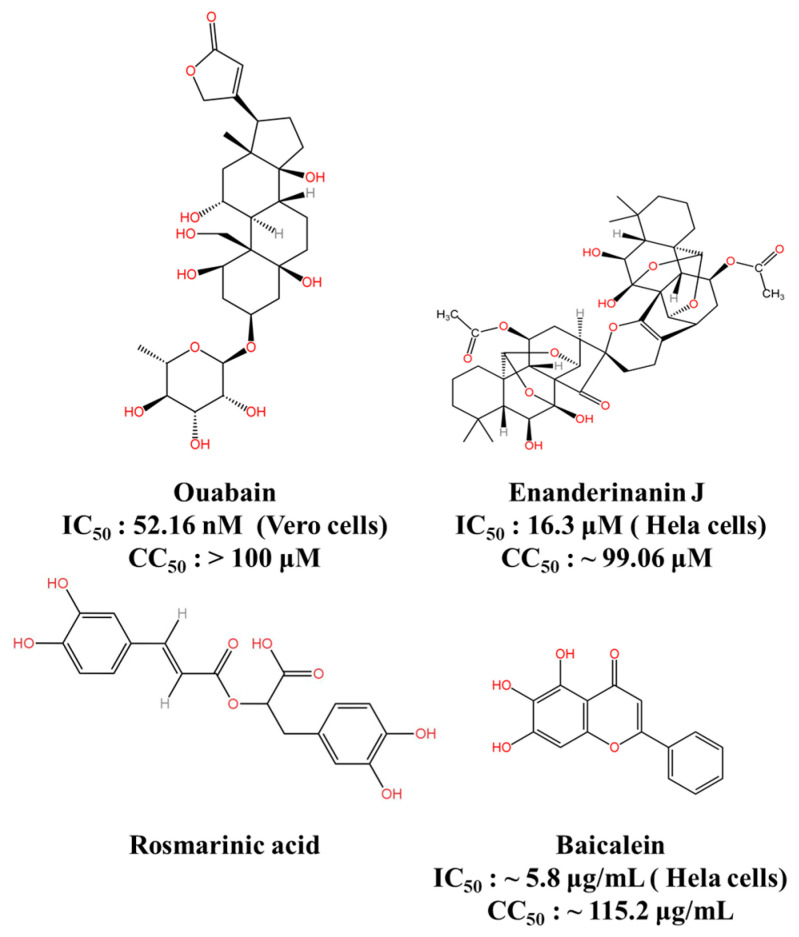
JEV inhibitors derived from natural products. This figure was originally created using KingDraw software 3.1.0.20.

Eight natural products were discovered to considerably inhibit JEV infection, with ouabain, a cardiac glycoside, proving to be the most promising candidate (Figure 7) [124]. Ouabain exhibited an IC_50_ of 52.16 nM in the inhibition of JEV. The antiviral effect of ouabain on JEV is positively correlated with extracellular NaCl but inversely correlated with KCl, suggesting that it exerts its antiviral effect via Na^+^/K^+^-ATPase. Furthermore, in a JEV-infected BALB/c mouse model, ouabain was observed to alleviate histopathological changes and reduce viral burden in the brain, resulting in protection against JEV-induced lethality in mice.

Enanderinanin J, a kaurane dimer, was extracted from *Isodon xerophilus* [125]. It significantly impeded JEV infection with an IC_50_ value of 16.3 μM and raised the levels of LC3-II and p62 in HeLa cells in both time- and concentration-dependent manners. Additionally, further research indicated that enanderinanin J restrained JEV infection by targeting autophagosome–lysosome fusion and alkalizing lysosome pH [126]. Furthermore, enanderinanin J demonstrated significant inhibition of DENV, ZIKV, and EV71 infections in host cells, with SI values of 6.9, 5.6, and 5.5, respectively. This indicates that the autophagy inhibitory activity of enanderinanin J protects host cells from these RNA viruses. Considering the promising anti-JEV activity of enanderinanin J, further studies could optimize its structure to enhance safety.

A study has presented proof that baicalein, a bioflavonoid, demonstrated remarkable antiviral effectiveness with an IC_50_ of around 5.8 μg/mL in Vero cells that were infected with JEV [127]. It was established that baicalein displayed potent direct virucidal activity (SI = 33.4) and anti-adsorption activity (SI = 15.8). Nevertheless, the mechanisms that underpin the antiviral influence of baicalein against JEV have not been defined due to research objectives being constricted. The virucidal activity of baicalein against extracellular JEV virions is potentially one of the mechanisms that explains the antiviral effects of this compound.

Rosmarinic acid is a phenolic compound found in Labiatae herbs and has anti-inflammatory properties [128]. In a study evaluating the efficacy of rosmarinic acid as a therapy against murine JE in 4- to 5-week-old BALB/c mice, it was found that rosmarinic acid treatment (25 mg/kg body weight for twice daily) had a significant effect on increasing the survival of JEV-infected mice, reducing the mortality rate to 20% [129]. In addition, rosmarinic acid abolished the increased expression of pro-inflammatory mediators. Both the antiviral and anti-inflammatory effects of rosmarinic acid were critical in reducing the severity of JEV-induced disease, suggesting that rosmarinic acid acts as an attractive antiviral agent against JE. This could lead to effective inhibitors with the potential for administration to JE patients.

Importantly, natural products and their derivatives provide significant benefits for treating viral infectious diseases. However, several obstacles must be overcome to develop a natural product [130]. The primary challenge is the fact that natural products can be used as drugs. Furthermore, hindrances may arise in sourcing and validating natural resources, enhancing the efficiency of functional natural substances and simplifying purification procedures. Consequently, the development of natural anti-JEV substances as medicinal drugs still requires substantial advancement.

### 6.5. Host-Directed Antiviral Agents

Antiviral drugs have generally been developed by directly targeting key viral components, but these strategies often fail due to the rapid emergence of drug-resistant viruses. Therefore, alternative antiviral approaches should be explored. As virus–host interactions play a critical role in viral life cycle and pathogenesis, host cell components are being considered as potential targets for antiviral therapeutic programmes [29,131]. 

**Figure 8:**
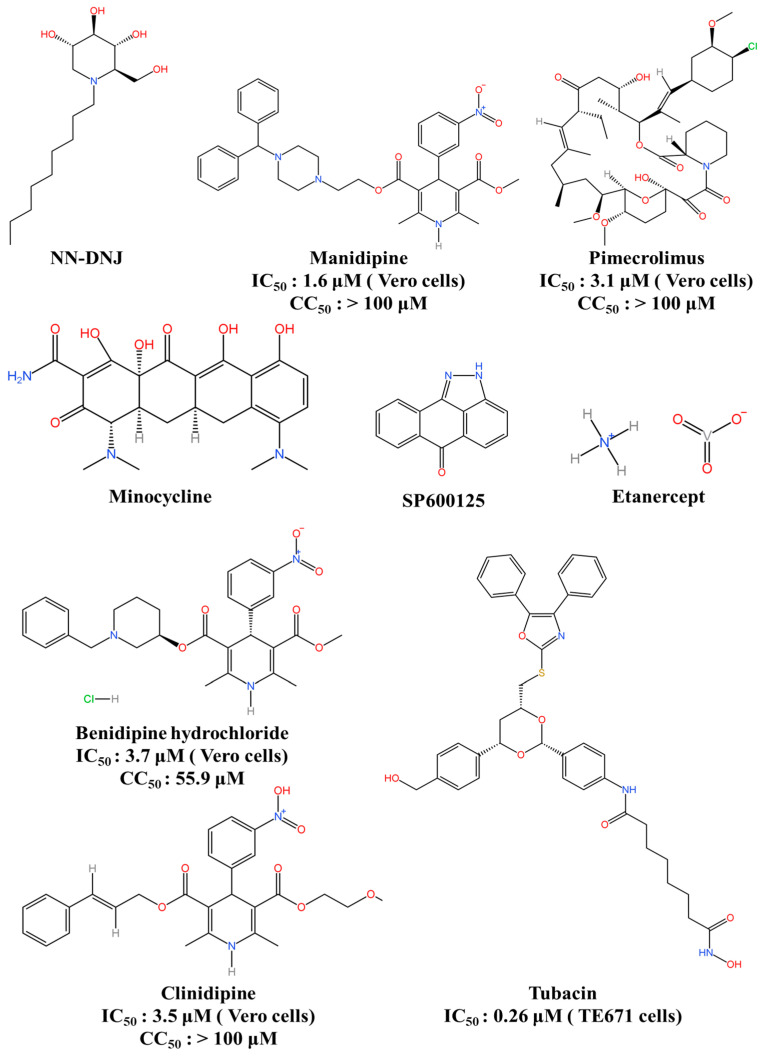
Host-directed JEV antivirals. This figure was originally created using KingDraw software 3.1.0.20.

JEV replicates in the cytoplasm and matures on the intracellular membranes of infected cells, budding from the ER and Golgi apparatus to secrete mature virions [3,132]. N-nonyl-deoxynojirimycin (NN-DNJ), a 9-carbon alkyl iminosugar derivative and an inhibitor of ER α-glucosidases [133], prevents the production of ER-budding JEV by blocking the trimming step of N-linked glycosylation. In a mouse model of JEV infection, the daily oral administration of NN-DNJ (200 mg/kg/day) decreased mortality in comparison to an untreated group. Moreover, it significantly elevated the survival rate up to 47%, and no evidence of sublethal illness was detected in the surviving mice (Figure 8) [132]. These findings indicate that NN-DNJ has the potential to suppress JEV infection both in vitro and in vivo. This proposed mechanism is probably the interference of JEV replication at the post-translational modification stage, leading to an indirect impact on the ER microenvironment. This study is a precursor for the development of alluring and innovative JEV inhibitors.

Wang et al. developed recombinant viral particles (RVPs) of JEV to select inhibitors via high-throughput screening and identified five blockades from the FDA-approved drug library. These hits encompass manidipine, cilnidipine, benidipine hydrochloride, pimecrolimus, and nelfinavir mesylate, all of which demonstrated inhibitory effects against JEV replication [134]. Among these, manidipine, a calcium channel inhibitor, has been utilised as a treatment for hypertension for an extended period [135]. Manidipine exhibited dose-dependent suppression of JEV RNA synthesis and a strong inhibition of JEV infection in multiple cells. The in vivo efficacy of this treatment was evaluated using a JEV-infected BALB/c mouse model, which showed that manidipine reduced the mortality rate to 20% and significantly decreased the viral load in infected mice compared to those treated with the vehicle. These results identified cytoplasmic calcium as a novel antiviral target for combating JEV infection, offering therapeutic opportunities for treating viral encephalitis induced by JEV.

Tumour necrosis factor alpha (TNF-α) has a decisive role in immunopathology within the central nervous system (CNS). An increased concentration of TNF-α in the serum and cerebrospinal fluid of JE patients poses a serious mortality risk [136]. The inhibition of TNF-α by competitive etanercept has proven to be an effective CNS injury treatment [137]. Ye et al. conducted an in vitro assay with neuron/glia cultures and observed that etanercept significantly reduced the inflammatory response caused by JEV infection. Furthermore, in vivo experiments demonstrated the effectiveness of etanercept treatment in reducing proinflammatory cytokine secretion, glial activation, and neuronal damage in JEV-infected mice. Etanercept treatment reduced the viral load in mouse brains and restored the impaired BBB, ultimately protecting the mice from JEV-induced lethality. The researchers demonstrated that the administration of etanercept rescued 50% of mice with a well-established JEV infection [138]. Considering the safety and antiviral activity of etanercept [138,139], it could be considered a therapeutic measure against neurotropic virus-induced viral encephalitis. Furthermore, there is potential for treating acute, established viral encephalitis with other clinically approved drugs, such as adalimumab and infliximab, which are commonly used to inhibit TNF-α [140,141].

Minocycline is a second-generation tetracycline that has been in use for over 30 years. It is a small molecule (495 kDa) that is highly lipophilic and capable of crossing the BBB [142,143]. Minocycline is a safe medication commonly prescribed for the prolonged treatment of infections, rheumatoid arthritis, and acne vulgaris [144]. An investigation into the antiviral effectiveness of minocycline utilised adult BALB/c mice (4–6 weeks) and a model of lethal challenge with the JEV strain GP78 [145]. Minocycline offered protection to JEV-infected mice when administered on day six following infection and after the onset of encephalitic symptoms. The treatment, given at a dose of 45 mg/kg body weight, effectively rescued 70% of the mice from JEV-induced death. Neuronal cell death, caspase enzyme activity, the activation of microglial cells, the production of pro-inflammatory molecules, and viral replication were significantly decreased following the administration of minocycline to mice infected with JEV on day nine after infection. Due to its antiviral properties, minocycline may be considered a potential treatment option for JE patients in human clinical trials. 

The SP600125 compound is a potent c-Jun N-terminal kinase1 (JNK1) inhibitor, competing with adenosine triphosphate in a reversible manner. It demonstrates exceptional selectivity compared to a range of kinases and enzymes, exceeding them by over 20-fold. In addition, SP600125 can penetrate cell membranes [146]. SP600125 was found to decrease the neuroinflammatory response and provide protection against encephalitis in a JEV-infected mouse model [147], demonstrating the crucial function of JNK1 signalling in JEV pathogenesis and proposing a potential aim for therapeutic intervention in JEV infection. 

Histone deacetylases (HDACs) and histone acetyltransferases (HATs) modify the acetylation of lysine residues in both histones and non-histone proteins [148]. HDACA6 is primarily located in the cytoplasm and has been shown to remove the acetylation from retinoic acid-inducible gene-I (RIG-I) during acute RNA virus infections. It is also involved in the RIG-I-dependent innate antiviral immune response [149]. Tubacin is a selective inhibitor of HDAC6, which shows concentration-dependent inhibitory effects on JEV-induced cytopathic and apoptotic processes. It causes a reduction in the Hsp90-NS5 interaction and viral proteins as well as antisense RNA genomes in infected cells. Additionally, it exhibits high potency in inhibiting JEV yield (IC_50_ = 0.26 μM) and significantly blocks the production of intracellular infectious virus particles [150]. Thus, tubacin exhibits significant potential as a host-targeting agent against JEV, demonstrating both preventive and therapeutic effects against JEV infection. 

In addition to the above-mentioned inhibitors of JEV, a study found that the ablation of Toll-like receptor 4 (TLR4) leads to a significant induction of systemic type I IFN innate responses and type I IFN expression and production from myeloid-derived cells during JEV infection. Knocking out TLR4 reduces the mortality rate of JE in mice [151]. Thus, it is worthwhile to investigate whether blocking the TLR4 pathway with antagonists like eritoran (an inhibitor that reduces inflammation and fibrosis in the livers of mice with chronic injury) [152] impacts the progression of JE by inducing innate type I IFN responses. This could potentially offer valuable insights into the advancement of treatment options for JEV-induced viral encephalitis.

## 7. Conclusions and Future Directions

JEV is a neurotropic virus that causes severe encephalitis in Southeast Asia and the Western Pacific; approximately 1.15 billion people are at risk of infection, and 10–15 thousand patients die each year [153,154]. JE remains a devastating disease, causing symptoms ranging from fever to severe encephalitis and death, with no specific treatment other than considerate, supportive care [155,156]. Current guidelines from the Centers for Disease Control and Prevention (CDC) suggest symptomatic and supportive measures as well as preventive strategies for the management of JEV infection. Control over JE suggests the important role of socio-economic parameters such as hygiene and education, but measures such as environmental manipulation, vector control, and changes in agricultural practices are difficult to achieve [3,157,158]. 

Various approaches are being employed to investigate potential antiviral agents against JEV infection. These include (i) a deliberate design based on the crystal structures of viral proteins; (ii) screening antiviral agent libraries approved by the FDA; (iii) the evaluation of recognised inhibitors of other human viruses; (iv) the chemical modification of viral inhibitors to enhance their therapeutic efficacy; and (v) intravenous treatment based on nucleic acids and immunoglobulins [55,158].

The amount of scientific literature published in recent years on JEV pathogenesis and therapeutic development is truly remarkable, and major advancements have been made in our knowledge of the pathology of JEV infection [3,14,19,35,55,158]. Novel therapeutic targets could offer additional opportunities for treating JE, while scientific advances in JEV research may promote treatment strategies for other flaviviruses. Table 2 outlines the antiviral strategies currently in development for JEV. However, only a limited number of JEV inhibitors in animal models have progressed to clinical trials, and the findings cannot be easily extrapolated to humans. Additionally, completing the entire drug development pipeline is challenging for many experimental antivirals. To ensure progress in scientific research, in vivo studies and clinical trials must be conducted promptly when appropriate.

Recently, peptide drugs have garnered significant attention due to their lower development costs and higher safety compared to small-molecule- and antibody-based antiviral drugs. However, peptides are burdened with certain drawbacks, such as relatively short half-lives and immunogenicity. To enable the clinical use of peptides as antiviral drugs, these drawbacks must be addressed in future studies [83]. Additionally, the immune system has been found to have both pathological and protective elements in relation to JE, indicating its paradoxical role. As a result, combination therapy trials incorporating both anti-inflammatory and antiviral drugs should be given due consideration. It is imperative to continue the search for and development of cost-effective therapies and immunisation strategies.

Successful trials of potential treatments, such as the JEV-specific monoclonal antibody, could provide clinical proof-of-concept for the development of treatments for other medically important arboviral encephalitides. Furthermore, this may also promote the development of treatments for emerging flaviviruses in the future, leading to more comprehensive treatment coverage during potential flavivirus epidemics [55,159].

Medicinal plants are an increasingly significant source of diverse natural compounds for drug discovery pipelines targeting viral infections. High-throughput drug screening at the cellular level is now a crucial tool in antiviral drug development. Various compound libraries provide access to hundreds of thousands of natural compounds, and through automated cell manipulation techniques and viral labelling, the identification of natural product candidates is rapid and efficient [123,124]. Initially, there are concerns regarding natural products, including absorption profiles, standardisation, uniformity, and stability, which should be considered. During the primary stages of drug discovery, it is critical to investigate the ADMET (absorption, distribution, metabolism, excretion, and toxicity) of compounds, which is essential in identifying those with poor absorption profiles [160].

Furthermore, recent developments in structural and molecular virology, as well as scientific methods for screening and studying inhibitors that target critical viral or host elements involved in the JEV life cycle, have been significant. Viral proteases are potential targets for therapeutic intervention due to their essential roles in viral life cycles. They are one of the earliest and most compelling examples of the effectiveness of structure-assisted drug design. Therefore, protease inhibitors should be given more prominence [161,162]. The path to host-directed antivirals for the treatment of JEV infection is promising, and investment in a deep understanding of the JEV life cycle, from viral attachment and entry to replication, maturation, and release at the molecular level, could also contribute to novel and effective treatment strategies. For example, the JEV NS3 and NS5 crystal structures’ availability has enabled the identification of possible inhibitor-binding locations to create the most effective drugs by using target-based drug development guided by structure [163,164]. Moreover, conducting in-depth analyses of the sequences and functions of JEV E proteins and NS proteins to identify the key sequences that affect viral protein function, as well as the recombinant expression of antiviral short peptide blockers based on these sequences, is a convenient method for the development of antiviral agents [165].

Importantly, other flaviviruses aside from JEV also pose a threat to populations, especially in areas with no prior infections. Recently, due to climate change and increased human mobility, these viruses have caused sudden outbreaks outside of their endemic areas [55]. Unfortunately, there is no specific therapy available for flavivirus infections, and, currently, there are only commercially available vaccines for three flaviviruses that are suitable for human use. Controlling mosquito and tick populations has proved arduous; the viruses they transmit are becoming increasingly important to human health, with few viable treatment options available [166]. Consequently, developing direct antivirals is challenging. Predicting which virus within the large flavivirus family will be responsible for the next epidemic is challenging. Therefore, host-directed antiviral agents may serve as a potential approach for pan-flavivirus agents [29]. This can be achieved by blocking host cell pathways that are essential for virus replication or for stimulating intrinsic antiviral programs that are shared among members of the virus family.

## Figures and Tables

**Table 1:** Target product profile (TPP) for a potential JEV drug.

	Optimum/Ideal	Minimum/Acceptable
Target population	Population presenting clinical symptoms of JEV infection in endemic areas	Population that recently visited an endemic area and presented a clinical manifestation of JEV infection
Efficacy	No circulating JEV in the bloodstream, as assessed by specific quantitative PCR assays	Eliminates or reduces the risks of the neurological impairment of patients
Administration and dosage	Single oral dose	The duration of treatment is extended throughout pregnancy; oral dose
Pharmacokinetic profile	Permeable to the blood–brain barrier	Repair of damaged blood–brain barrier
Safety and tolerability	Safe to be taken during pregnancy (category A ^a^)	Safe to be taken during pregnancy (category B ^b^)
Adverse reactions	No observed adverse reaction	Reversible and mild adverse reactions
Drug interactions	No drug interactions	No interaction with common drugs against depression, diabetes, and high blood pressure during pregnancy
Contraindications	No contraindications	/
Precautions and warnings	No precautions or warnings	No teratogenicity and genotoxicity
Storage and handling	Stable for 1 year at room temperature	Require refrigeration (−20 °C) for stability
Cost of goods	Less than USD 5 per treatment course	Less than USD 100 per treatment course

^a^ Category A: well-controlled and solid studies have failed to reveal a risk to the foetus in the first trimester of pregnancy, and there is no definite evidence of risk in later trimesters. ^b^ Category B: animal reproduction studies have failed to reveal a risk to the foetus, and there are no well-controlled and solid studies in pregnant women.

**Table 2:** Summary of antiviral strategies developed for JEV.

Drug Target	Compound/ Drug Name	Mechanism of Action	In Vitro Activity: IC_50_ or EC_50_ and CC_50_ or LD_50_ (Utilised Cell Line)	In Vivo Efficacy: % Survival (Explored Animal Model)	References
Entry Inhibitors	P1	Interacts with the JEV E protein	IC_50_: 35.9 μM (BHK-21 cells) CC_50_: >100 μM	70% (C57/BL6)	[83]
	Berbamine	Decreases LDLR levels at the cell surface	IC_50_: 1.62 μM (A549 cells) CC_50_: ~126 μM	75% (BALB/c)	[84]
	BP34610	A broad-spectrum flavivirus inhibitor that may target the flavivirus E protein	ND * CC_50_: ~94.55 μM	ND	[88]
	RC-101	Targets the DE loop of the E protein	IC_50_: 10.67 μM (BHK-21 cells) CC_50_: >100 μM	ND	[38]
	MG132	Interferes with JEV intracellular trafficking	ND * CC_50_: >3.2 μM	ND	[95]
	Lactacystin	Interferes with JEV intracellular trafficking	ND * CC_50_: >25.6 μM	ND	[95]
	Cur-CQDs	Binds to the JEV E protein	IC_50_: 0.9 μg/mL (BHK-21 cells) CC_50_: >144 μM	ND	[98]
RdRp Inhibitors	2′-C-MeC	Inhibits JEV replication	EC_50_: ~0.66 μM (Vero cells) CC_50_: >200 μM	ND	[108]
	DMFA	Inhibits JEV replication	EC_50_: ~0.85 μM (Vero cells) CC_50_: >200 μM	ND	[108]
Protease Inhibitors	CW-33	Targets JEV NS2B-NS3	pIC_50_: 5.09	ND	[116]
	Andrographolide	Inhibits JEV NS3 protease	IC_50_: 2 μg/mL	ND	[117]
	Abscisic acid	Binds with JEV NS2B-NS3 protease	IC_50_: 100 μg/mL LD_50_: 4260 mg/kg	ND	[48]
	Aloe-emodin	Binds with JEV NS2B-NS3 protease	IC_50_: 7.3 μg/mL LD_50_: 435 mg/kg	ND	[48]
Bioactive Natural Products and Their Derivatives	Ouabain	Targeting the Na^+^/K^+^-ATPase	IC_50_: 52.16 nM (Vero cells) CC_50_: >100 μM	67% (BALB/c)	[124]
	Enanderinanin J	Inhibits autophagosome–lysosome fusion	IC_50_: 16.3 μM (Hela cells) CC_50_: ~99.06 μM	ND	[126]
	Baicalein	Virucidal activity; inhibits adsorption activity	IC_50_: ~5.8 μg/mL (Hela cells) CC_50_: ~115.2 μg/mL	ND	[127]
	Rosmarinic acid	Anti-inflammatory effect	ND *	80% (BALB/c)	[129]
Host-Directed Antiviral Agent	NN-DNJ	Inhibits α-glucosidases enzymes	ND *	54% (ICR)	[132]
	Manidipine	Targets NS4B and calcium channel	IC_50_: 1.6 μM (Vero cells) CC_50_: >100 μM	80% (BALB/c)	[134]
	Cilnidipine	Inhibits calcium channel	IC_50_: 3.5 μM (Vero cells) CC_50_: >100 μM	ND	[134]
	Benidipine hydrochloride	Inhibits L-, N-, and T-type calcium channels	IC_50_: 3.7 μM (Vero cells) CC_50_: 55.9 μM	ND	[134]
	Pimecrolimus	Inhibits inflammatory cytokine secretion	IC_50_: 3.1 μM (Vero cells) CC_50_: >100 μM	ND	[134]
	Etanercept	Inhibits the downstream signalling pathways of TNF-α	ND *	50% (BALB/c)	[138]
	Minocycline	Inhibits inflammatory cytokines and active caspase 3 activity	ND *	70% (BALB/c)	[145]
	SP600125	Inhibits JNK signalling	ND *	40% (BALB/c)	[147]
	Tubacin	Inhibits histone deacetylases	IC_50_: 0.26 μM (TE671 cells)	ND	[150]

ND *: in vitro efficacy was assessed via a viral inhibition assay, although IC_50_ and EC_50_ were not measured; ND: efficacy in vivo was not evaluated; CC_50_: 50% cytotoxic concentration; LD_50_: semilethal concentration.
